# The COVID-19 pandemic and N95 masks: reusability and decontamination methods

**DOI:** 10.1186/s13756-021-00921-y

**Published:** 2021-05-29

**Authors:** Alexandra Peters, Rafael Palomo, Hervé Ney, Nasim Lotfinejad, Walter Zingg, Pierre Parneix, Didier Pittet

**Affiliations:** 1 Infection Control Programme, University of Geneva Hospitals and Faculty of Medicine, 4 Rue Gabrielle-Perret-Gentil, 1211 Geneva 14, Switzerland; 2 University of Geneva, Geneva, Switzerland; 3 Department of Research, Faculty of Medicine, Mashhad University of Medical Sciences, Mashhad, Iran; 4 Nouvelle Aquitaine Healthcare-Associated Infection Control Centre, Bordeaux University Hospital, Bordeaux, France

**Keywords:** COVID-19, Face masks, Mask reuse, Disinfection, Sterilization, N95, FFP2, Infection prevention, Healthcare environmental hygiene, IPC

## Abstract

**Background:**

With the current SARS-CoV-2 pandemic, many healthcare facilities are lacking a steady supply of masks worldwide. This emergency situation warrants the taking of extraordinary measures to minimize the negative health impact from an insufficient supply of masks. The decontamination, and reuse of healthcare workers’ N95/FFP2 masks is a promising solution which needs to overcome several pitfalls to become a reality.

**Aim:**

The overall aim of this article is to provide the reader with a quick overview of the various methods for decontamination and the potential issues to be taken into account when deciding to reuse masks. Ultraviolet germicidal irradiation (UVGI), hydrogen peroxide, steam, ozone, ethylene oxide, dry heat and moist heat have all been methods studied in the context of the pandemic. The article first focuses on the logistical implementation of a decontamination system in its entirety, and then aims to summarize and analyze the different available methods for decontamination.

**Methods:**

In order to have a clear understanding of the research that has already been done, we conducted a systematic literature review for the questions: what are the tested methods for decontaminating N95/FFP2 masks, and what impact do those methods have on the microbiological contamination and physical integrity of the masks? We used the results of a systematic review on the methods of microbiological decontamination of masks to make sure we covered all of the recommended methods for mask reuse. To this systematic review we added articles and studies relevant to the subject, but that were outside the limits of the systematic review. These include a number of studies that performed important fit and function tests on the masks but took their microbiological outcomes from the existing literature and were thus excluded from the systematic review, but useful for this paper. We also used additional unpublished studies and internal communication from the University of Geneva Hospitals and partner institutions.

**Results:**

This paper analyzes the acceptable methods for respirator decontamination and reuse, and scores them according to a number of variables that we have defined as being crucial (including cost, risk, complexity, time, etc.) to help healthcare facilities decide which method of decontamination is right for them.

**Conclusion:**

We provide a resource for healthcare institutions looking at making informed decisions about respirator decontamination. This informed decision making will help to improve infection prevention and control measures, and protect healthcare workers during this crucial time. The overall take home message is that institutions should not reuse respirators unless they have to. In the case of an emergency situation, there are some safe ways to decontaminate them.

## Introduction

In the context of a pandemic caused by SARS-CoV-2, many healthcare facilities are lacking a steady supply of masks. Currently, global demand is so high that suppliers cannot meet it. Many countries are affected at the same time, resulting in high demand, low production and interrupted delivery chains. As COVID-19 spreads over the globe, it is increasingly affecting developing countries with limited resources for public health. This is posing some serious challenges to managing the spread of the pandemic and to providing care to people affected. Protecting the health of the nursing staff is paramount in this fight, and it is essential to find viable alternatives to the scarcity of masks. This emergency situation warrants the taking of extraordinary measures to minimize the negative health impact from an insufficient supply of masks [1]. The decontamination and reuse of healthcare workers’ (HCWs’) N95/FFP2 masks is a promising solution [2–6].

This approach is currently being explored in a number of countries around the world, and an increasing number of decontamination methods are being studied and analyzed in the scientific literature. Some studies have shown positive and convincing results, suggesting that the reuse of disposable masks can be a safe and implementable temporary solution to address the shortage of masks during the current pandemic [6–9].

Before choosing the method for decontamination, a number of elements must be taken into account. A large part of the process of safely decontaminating and reusing masks has nothing to do with the decontamination process at all. Simply the process of safely collecting masks, decontaminating them, and getting then back safely to the HCWs, requires the establishment of a complex internal logistics system that is not detailed in most articles. A circuit must be put in place between the HCW giving their used mask for decontamination and the HCW recovering a sterilized mask ready for use. In addition, because of the numerous opportunities for damaging the integrity or function of the mask as well as the inherent risks of contamination, setting up this collection and redistribution system is just as important as the decontamination itself.

This article has two objectives. The first is to focus on the logistical implementation of such a system in its entirety. For this, we relied heavily on the results and advice from our laboratory at the University Hospitals of Geneva and Faculty of Medicine (HUG), Geneva, Switzerland, and our partner laboratories. The second is to summarize and analyze the different available methods for decontamination, using both the data available in the literature, as well as some yet unpublished data. The approved methods are scored according to a number of variables: efficacy, risks, cost, time, complexity, and reusability. The overall aim of the article is to provide the reader with a quick overview of the various methods for decontamination and the potential issues that need to be taken into account when deciding to reuse masks. We are convinced that this will help people make informed decisions for their own institutions. The only masks referred to in this article are the FFP2/N95 type, and we do not make any recommendations concerning other types of masks.

## Methods

Our paper aims to look at the practical aspects of implementing mask decontamination as well as the individual methods. The logistics of mask selection, collection, and redistribution are crucial, no matter which decontamination method is chosen, and we have detailed this process in the discussion section.

For the sake of clarity, we will use the word "decontamination" to mean either disinfection or sterilization in this first part of the article. We will distinguish between the methods of sterilization and the methods of disinfection in the “Methods of disinfection and sterilization” section.

In order to have a clear understanding of the research that has already been done, we conducted a systematic literature review (“Decontaminating N95/FFP2 masks for reuse during the COVID-19 epidemic: a systematic review”) which is published separately. This review aimed to find out what the tested methods for decontaminating N95/FFP2 masks were, and what impact do those methods have on the microbiological contamination and on the physical integrity of the masks. We used the papers that were included, as well as some of the papers that were excluded but useful, for example which may have tested fit and filtration of masks post decontamination, but where the outcome wasn’t a microbiological assessment of the decontamination. We used the results from our laboratory and from the systematic review to inform the section of the paper on the different available methods and the analyses of these methods according to the variables defined in the introduction.

We analyzed all of the methods that successfully sterilized or disinfected the masks studied, without being detrimental to their fit or filtration capabilities. We briefly mention the unsatisfactory methods in the “Others” section. The search strategy is accessible on: https://www.crd.york.ac.uk/PROSPEROFILES/193309_STRATEGY_20200619.pdf

## Results

The literature is quite poor currently, with only 35 published studies looking at the microbiological results of different methods of decontamination of the respirators. Still, there is starting to be a major growth in publications around this subject that are looking at all aspects of mask reuse from logistics, to healthcare worker acceptance, to costs and effects on the physical characteristics of the masks.

**Fig. 1:**
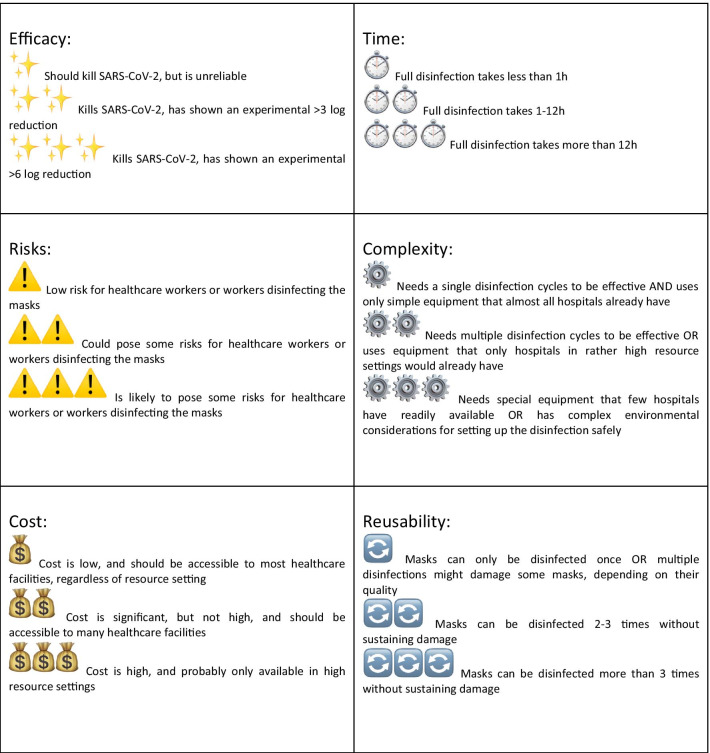
Visual guide for reprocessing and reuse of respirators using emojis

Table 1 shows criteria for excluding the reprocessing and reuse of masks according to the parties concerned. Figure 1 summarizes a visual guide for reprocessing and reuse using emojis. Our results are detailed in Table 2. UVGI, hydrogen peroxide, steam, ozone, ethylene oxide, dry heat and moist heat were the methods that were deemed sufficiently satisfactory for consideration by healthcare institutions.

**Table 1:** Reprocessing and reuse of N95 masks: exclusion criteria, organized by the parties concerned

Population	Exclusion criteria
For healthcare workers	Model other than N95 or FFP2
	Presence of stain(s) or soil of any type
	Any visible damage to the mask
	Any alteration of how the mask attaches/ is held in place
	Any deformation or physical alteration of the mask
	Maximum number of sterilization cycles reached (according to inscription on the mask)
For the personnel in charge of preparing the masks for disinfection/reprocessing	All of the preceding criteria
	Presence of mold/mildew (either visible or by odor)
	Inadequate labeling: absence or illegibility of labeling on the mask, present on the wrong part of the mask/written with the wrong type of marker
	Presence of hair on the mask
For the personnel in charge of organizing and sorting the masks after disinfection OR for the healthcare workers receiving the masks after disinfection	All of the preceding criteria
	Any uncertainty of who the mask belongs to
	Any uncertainty concerning the number of disinfection cycles

**Table 2:** Table of different decontamination methods according to the defined variables (see Fig. 1 for scoring with emojis.)

*Dry heat*	
Efficacy to	The decontamination method at a temperature of 65 to 70 °C shows a > 3 log reduction of most microorganisms tested, though not all of them (see Table 3). [7, 38–42]. Initial sorting of the masks must be performed very carefully, as dry heat treatments tend to fix proteins and organic matter to the mask material [43]. In other words, the presence of even an almost invisible stain means that the mask must be disposed of. Double exposure to heat over a long period of time can achieve decontamination values similar to those obtained with water vapor at 121 °C [7]
Risk	The exposure to dry heat does not cause the deposition of harmful molecules. It is therefore safe for HCWs. No other precautionary measure should be taken in addition to those related to the handling of potentially infected masks for personnel. The machines used must have an efficient and appropriate ventilation systems with an external exhaust from the room in which they are being treated (which is normally the case for rooms with this type of equipment) [7]. Using this method, and because the masks are only disinfected, it is essential that decontaminated masks are distributed to their original owner for the reasons mentioned in the "Efficacy" section
Cost to	No cost estimates have been provided for this process, but the basic equipment is not expensive. For a two-step process, costs may be higher due to the need for two different types of devices as well as the added manpower to transport masks between the first and the second sterilization stages
Time to	The duration of a disinfection cycle is less than an hour. The masks are treated in the disinfection device for 30 min and are ready for use shortly after. No desorption, evaporation or drying period is necessary. Personnel simply have to wait until the temperature of the masks is cool enough to handle safely before distributing them. For a two-step process, time is significantly longer
Complexity	Drying cabinets, laboratory drying rooms, ovens or washer-disinfectors are the devices offering the possibility of disinfecting the masks with dry heat. Their presence is probably common in high-resource hospitals. One cycle is enough to effectively eliminate SARS-CoV-2 and most other microorganisms
Reusability	Two disinfections per mask are recommended, resulting in three uses in [7, 44]. This method maintains the filtering functions and the physical integrity of the masks [38–41]
*Ethylene oxide*	
Efficacy	This process eliminates SARS-CoV-2 as well as other microorganisms that may be present on the mask [15, 21]. Experimental study has shown a > 6 log reduction of microorganisms tested [20]
Risk	Several articles in the literature exclude the use of ethylene oxide because of its toxicity. This gas is known to be carcinogenic and teratogenic if the wearer is subjected to chronic inhalation [2]. Following the proper procedures should, however, avoid this problem. In Geneva, the experiments carried out concluded that this method needed to include a 48 h desorption time to guarantee the absence of harmful particles in the masks [4, 11]. It is extremely important to respect this desorption time before using the masks. The personnel in charge of sterilization must avoid any exposure to gas and handle the used masks with gloves and mask. As ethylene oxide is a flammable and explosive gas, safety measures associated with this risk must be considered. An additional risk factor could be that if the masks need to be reprocessed at a site external to the hospital, there could be inherent issues with oversight and quality control
Cost to	The price of autoclaves, the necessary equipment and the cost of the workforce has to be considered. The costs have not been estimated in the included studies. However, there is a need for a specific external structure (see section "Complexity") and trained workforce, which can make the process less accessible. According to the Swiss pilot plan, a cycle of 30 masks requires 10 min of direct labor, meaning 20 s per mask [14]. As this method of sterilization is often used in industry it may, be feasible to perform at a large scale, which could reduce the cost per mask quite significantly. The cost of 30 masks sterilized in the HUG is 86 CHF (€80) which means 2.60 CHF per mask (€2.40). In an industrial process in Switzerland, 8000 masks can be sterilized for 2500 CHF (€2325 which means 0.30 CHF per mask (€0.28)
Time	The total time needed for sterilization is around 51 h. The sterilization process itself lasts for 3 h. It is necessary to wait an additional 48 h before handling or reusing the masks. According to the HUG project, no residue of ethylene oxide was present on the tested masks after the 48 h desorption time. It is very important for the health of personnel that the desorption of ethylene oxide is complete. Additional time may be added in the case of the reprocessing needing to take place in a structure external to the healthcare facility
Complexity	The infrastructure and equipment necessary for ethylene oxide sterilization is lacking in most hospitals. Thus, a dedicated worker external to the care center is necessary for the implementation of this method. This complicates the recycling process, as the masks must leave the medical center, be sterilized and then brought back. This particular sterilization technique is most often implemented in industrial processes, which may also imply a greater sterilization capacity. The cycles carried out in Switzerland contained 30 masks at a time, which is advantageous. One cycle is sufficient to guarantee complete sterilization of the masks
Reusability	Up to three sterilizations can be performed, resulting in a maximum of four uses for each mask [45]. This method does not affect the filtering properties or the physical integrity of the mask [2, 46]
*Hydrogen peroxide (gaseous)*	
Efficacy	This process eliminates SARS-CoV-2 as well as any other microorganisms that might be present on the mask [21, 40, 42, 47–49]. Experimental study has shown a > 6 log reduction of microorganisms tested [20, 50, 51]
Risk	The personnel in charge of sterilization is no more at risk than for another disinfection process using an autoclave. Wearing a mask and using gloves are recommended. After placing in the sterilizers, the masks must remain at least one hour in order to guarantee the full evaporation of the hydrogen peroxide [14] and personnel must be kept from breathing it in when opening the autoclave. For HCWs as well, it is important that the evaporation time of the hydrogen peroxide vapors is respected before handling or reusing the mask. Following this, the masks can be used again. The residual quantity of the decontaminant is below the permissible exposure limit (PEL) [52, 53]
Cost	The price of autoclaves, the necessary equipment and the cost of the workforce has to be considered. The Swiss pilot plan calculated a price of 15 CHF (€14) per sterilized mask [12]. It is also estimated that a cycle requires 10 min of labor, which amounts to one minute per mask (as most of the tested autoclaves had a capacity of 10 masks per cycle)
Time	The duration of a sterilization cycle varies between 1 h 24 min and 2 h depending on the program used. The hydrogen peroxide vaporization programs themselves last 24–55 min [22, 31]. The masks must then rest for one hour to allow the hydrogen peroxide to evaporate for the reasons mentioned previously in the "Risks" section
Complexity	A single passage in the autoclave guarantees sterilization of the masks. However, autoclaves compatible with hydrogen peroxide sterilization are rarely present in healthcare facilities. The adoption of this method would probably imply the acquisition of this equipment. The presence of a zone dedicated to the safe evaporation of hydrogen peroxide is also necessary. On the other hand, these programs are effective for 10 to 20 masks at a time [4, 11]
Reusability to	The number of sterilization cycles that a mask can withstand without damage varies from between 2 to 10 cycles depending on the model of autoclave used. As long as the masks have not reached their maximum number of possible decontaminations, their filtering capacities and integrity are maintained [4, 9, 46]. When they reach the maximum number, they must be discarded The hydrogen peroxide vapor sterilization systems proposed by the Battelle Memorial Institute and the article by Bergam et al. served as references for the FDA and the CDC [2, 54, 55]. The method proposed by Battelle is interesting as it allows up to 20 sterilizations per mask [3, 8]
*Moist heat*	
Efficacy	This process eliminates SARS-CoV-2 as well as any other microorganisms that might be present on the mask [23, 56–58]. Moist heat can be generated by different ways. Experimental study of microwave-generated steam has shown a > 6 log reduction of microorganisms tested [24]. Moist heat disinfection in an oven has shown a > 3 log reduction of microorganisms tested [59, 60]. Moist heat disinfection in a rice cooker has shown a > 3 log reduction of microorganism tested [61]
Risk	The exposure to steam does not cause the deposition of harmful molecules. It is therefore safe for HCWs. No other precautionary measure should be taken in addition to those related to the handling of potentially infected masks for personnel
Cost	The devices used (microwave and bags, rice cooker, containers and oven) are not expensive compared to other techniques. This accessibility is a significant advantage for low resource environments
Time	The masks undergo a cycle close to a minute in the microwave and then dry for a maximum of an hour. In one experiment, the masks were dried for 30 min or 60 min under conditions of approximately 20 °C and 60% relative humidity [23]. The total time for a disinfection cycle should be approximately one hour. For moist heat generated by a rice cooker, time of treatment is also less than an hour [61]. The disinfection process lasts 15 min and masks must be dried after. For moist heat in an oven, masks are exposed during 20 to 30 min. However, the masks are located in a container during the process of disinfection that must have been heated before. This process can request 3 h of preheating the containers without having a mask in them [59, 60]
Complexity	A single pass in the microwave, rice cooker or oven guarantees elimination of the microorganisms tested [23, 24]. The complexity of implementing this method is low as the necessary equipment is accessible. Furthermore, the MSB X/Y bags for microwave-generated steam can be used as storage bags before the disinfection [23]. The capacity of the microwaves, ovens or rice cookers can be a limiting factor
Reusability	For microwave-generated steam, masks can be disinfected more than 3 times. In one study masks’ fit and function were preserved after 20 cycles [24]. No information about reusability is available for oven moist heat and rice cooker moist heat disinfections. However, one disinfection by these two methods does not affect the filtering properties or the physical integrity of the masks [59–61]
*Ozone*	
Efficacy	All of the studies showed that the ozone disinfected well, and has the potential for sterilization [26, 62, 63]
Risk	Ozone is toxic and a lung irritant but unstable; it degrades into oxygen. With adapted equipment and ventilation, this method is low risk, but system needs to be functioning well without leaks, etc [64]
Cost	Dedicated equipment and oxygen gas is needed to produce the ozone on site, but otherwise this method is not excessively expensive [65]
Time	Relatively short time is needed, tests on masks were conducted with exposure times of between 5 min and two hours [25, 74, 75]
Complexity	Some specialized equipment and a sealed chamber for the masks is needed
Reusability	The only area of the facemasks that seemed to have an issue is the straps, which also puts into question the usability of ozone as a successful decontamination in the first place [62, 63]. In order to recommend this treatment, masks must be verified for compatibility with this method
*Steam sterilization*	
Efficacy	This process eliminates SARS-CoV-2 as well as other microorganisms that may be present on the mask [35, 66]. Experimental study has shown a > 6 log reduction of microorganisms tested [20]
Risk	The exposure to steam does not cause the deposition of harmful molecules. It is therefore safe for HCWs. No other precautionary measure should be taken in addition to those related to the handling of potentially infected masks for personnel
Cost	The costs depend mainly on the presence or absence of the water vapor sterilizers and their capacity
Time	The duration of the process until the masks can be is usually less than 1 h. The sterilization programs themselves last 15–20 min. The masks go out dry so they can be used directly after their sterilization
Complexity	One sterilization cycle is sufficient for the mask to be used again. Steam sterilizers are found in the majority of sterilization units in healthcare facilities, though there is a need to accommodate for a drying area for masks. One cycle is sufficient to guarantee sterilization of the masks
Reusability	The number of sterilization cycles varies by country. The Austrian Ministry of Health recommends a single sterilization and therefore two possible uses per mask. In the Netherlands, health authorities state that two sterilization cycles are feasible (resulting in 3 uses per mask) [7]. Concerning the filtration properties of masks after sterilization, a Dutch study indicated that these do not vary significantly between masks sterilized once and 5 times. However, there is nonetheless a decrease in filtration capacities after sterilization [67]
*Ultraviolet Germicidal Irradiation (UV-C)*	
Efficacy	This process eliminates SARS-CoV-2 as well as any other microorganisms that might be present on the mask. Most of studies have shown a > 3 log reduction of microorganisms tested. [6, 29, 35, 58, 68–74]. Two studies have shown a > 6 log reduction of microorganisms tested [6, 29]. It is generally accepted that the efficacy of disinfection by UV strongly depends on the dose administered and thus on the intensity of the lights available [2, 75, 76]. This method destroys SARS-CoV-2 if a sufficiently high-energy UV apparatus is used. According to laboratories in the Nebraska [6], 2–5 mJ/cm^2^ are necessary for the inactivation of the coronavirus. In practice, they recommend not exposing the masks to any less than 300 mJ/cm^2^. The study showed that the bacterial and viral load was reduced by 6 logarithms when subjected to 60–300 mJ/cm^2^. A study from Switzerland concludes that a 60 mJ/cm^2^ UV irradiation is sufficient to reduce by > 3 log of virus [68]. The efficacy also depends on the sufficient exposure of the mask material to the light. It is very important that the entire surface of the masks is reached by light, either directly or by reflectors, which can pose certain challenges to implementation. The efficacy depends also of the distance between the lamp and the mask and time of exposure [77]
Risk	The exposure to UV does not cause the deposition of harmful molecules [52, 53]. It is therefore safe for HCWs. In addition to the protective measures linked to the handling of used masks, the personnel in charge of disinfection must of course avoid any exposure to the UV rays. It is therefore recommended to dedicate a room to the process, and to turn on the lamps remotely after the room has been closed
Cost	The initial cost of buying the UV machine is quite high. UV lamps and all the material necessary for the reflection of light are expensive. However, the cost per cycle is much lower than the one for HPV sterilization if a high number of cycles are performed. It is, however, still a method mainly accessible to healthcare facilities in high resource environments. It is also worth noticing that the cost varies according to the quality of the installation
Time	A disinfection or sterilization cycle (> 3 log reduction or > 6 log reduction) lasts less than one hour. The exposure time required is between 15 and 40 min and no additional steps are necessary [6]. Calculations must be made to ensure that the distance between the lamp and the mask is correct. The time needed for this type of decontamination increases as the mask is placed further form the source [77]. Time of sufficient exposure depends also on the intensity of the lights as said before
Complexity	This method uses equipment which is not present in most hospitals. Moreover, it requires the installation of a room intended solely for decontamination. One cycle is enough to effectively eliminate SARS-CoV-2 and most other microorganisms
Reusability	The possible number of cycles for each mask remains to be defined. According to one study, masks exposed to 1 J/cm^2^ can be reprocessed up to 10 times without the filtering properties or the integrity of the mask being affected [5, 44]. This same study also concludes that the exposure for the majority of models of N95 masks with 20 cycles causes almost no alteration, though some models of masks showed damage or dysfunction One study tested UV disinfection followed by dry heat disinfection (65 °C). This method could be considered if the UV lamps available are of low intensity, as the dry heat further reduces the viral and bacterial load [78]

The criteria are as follows: efficacy of the method of disinfection, risks for HCWs and for the staff in charge of disinfection, costs involved, time required for a complete cycle, complexity of the process and possibility of sterilizing the masks several times (while maintaining their properties). This assessment is arbitrary. The symbols used in this paper and their meaning are detailed in Fig. 1. The comparison of the different disinfection/ sterilization methods is shown in Table 2.

Our systematic review found one experiment each with Benzalkonium Chloride wipes, Non-antimicrobial detergent wipes, peracetic acid dry fogging system (PAF), UVA, and a combination of UVGI with medium humidity heat. It found two experiments for each of the methods using Ethylene oxide, hypochlorite, gaseous hydrogen peroxide with peracetic acid, and UVGI with dry heat, and three each using ozone, steam and ethanol.

We found nine studies that looked at decontamination with dry heat, 11 that tested gaseous hydrogen peroxide, 13 studies using moist heat, (five of which were generated using a microwave) and 15 that looked at UVGI decontamination (see Table 3).

**Table 3:** Results of the systematic review

Method	# Studies	Type of pathogen	Microbiological result	Integrity, fit and filtration
Benzalkonium chloride wipes [30]	1	Bacteria	Disinfection	Failed
Dry heat [35, 38–42, 61, 74, 79]	9	Viruses and Bacteria	Disinfection and failure	6 studies: all passed
Ethanol [35, 40, 48]	3	Viruses and spore-forming bacteria	Disinfection and failure	Failed
Ethylene oxide [20, 79]	2	Viruses	Sterilization and disinfection	2 studies: all passed
Gaseous hydrogen peroxide [20, 40, 42, 47–49, 51, 79, 80]	11	Viruses, bacteria, spore-forming bacteria, fungus	Sterilization, disinfection, 1 failure	9 studies: all passed
Gaseous hydrogen peroxide with peracetic acid [74, 81]	2	Viruses, bacteria and spore-forming bacteria	Sterilization	1 study: passed
Hypochlorite [30, 35]	2	Bacteria and spore-forming bacteria	Disinfection	1 study: failed
Moist heat [39, 47, 57–61, 79,]	8	Viruses, Bacteria, spore-forming bacteria	Sterilization, disinfection, failure	5 studies: passed
Microwave-generated moist heat [23, 24, 58–60]	5	Viruses and Bacteria	Disinfection	3 studies: passed, 1 study: mixed results
Non-antimicrobial detergent wipes [30]	1	Bacteria	Failure	Failure
Ozone [26, 62, 63]	3	Viruses and Bacteria	Sterilization, disinfection, 1 failure	3 studies: 2 failed elastic band but passed face piece, 1 study: passed
Peracetic acid dry fogging system (PAF) [20]	1	Viruses	Sterilization and disinfection	Passed
Steam [20, 35, 66,]	3	Viruses, bacteria and spore-forming bacteria	Sterilization, disinfection, failure	2 studies: passed
UVA [35]	1	spore-forming bacteria	Failure	N/A
UVGI [29, 35, 40, 42, 48, 58–60, 69, 70, 72, 74, 79, 82, 83]	15	Viruses, bacteria, spore-forming bacteria, fungus	Sterilization, disinfection, failure	7 studies: 1 failure, 1 partial failure, 5 passed
UVGI + dry heat [68, 79]	2	Viruses and Bacteria	Disinfection and failure	Passed
UVGI + medium humidity heat [79]	1	Viruses	Sterilization, disinfection, failure	Passed

## Discussion

### Collection and distribution: a circular system

Although the literature that has emerged in response to COVID-19 has paid close attention to the different methods of mask decontamination, it is poor in recommendations concerning the collection and distribution system that this process inevitably involves. Decontamination of masks is only useful if part of an effective and safe system for collecting, decontaminating and distributing the masks. It is therefore important to detail the various stages, actors and selection criteria necessary for the proper functioning of such a system.

### Mask selection and exclusion criteria

Usually, a medical device is washed and disinfected before sterilization can take place, as this ensures safety if a device is used for different patients or HCWs. If these preliminary steps are not possible (as is the case with mask reprocessing), then collection and distribution must be individualized because safety cannot be guaranteed. Even if the decontamination process is effective for SARS-CoV-2, it may not be for spores or mycobacteria, especially in the presence of soil that might not be visible to the personnel inspecting the masks.

Before speaking about the different methods of decontamination and their individual qualities or challenges, it is important that we discuss selection and exclusion criteria for the masks. A selection filter must be applied so that only eligible masks undergo decontamination. As mentioned previously, not all masks can be reused (Table 1). It is important to mention that selection of criteria is to a certain extent arbitrary. We based ourself on the existing methods, which does not mean every criteria is scientifically proved necessary.

Selection begins with the people who are wearing the mask. The clarification of the selection criteria must be understood by the users and carried out with them. This step is crucial because any error at this level could compromise the process. It is also important to note that the quality of the masks can vary a great deal from one manufacturer to the next and may affect the results of the decontamination process. Some decontamination methods can only be performed with high quality masks. If this is the case for a specific decontamination method, this information will be detailed in the section that speaks about the individual methods.

The Swiss Society for Hospital Sterilization (SSSH) and their European counterparts agree that the masks must be free from visible soil or stains [4, 13]. These can include blood, nasal secretions or other bodily fluids, or even the stain of lipstick. In these cases, the mask must be discarded; it cannot be decontaminated and reused. Likewise, any visible damage to the mask or deterioration of the support system (elastic bands), also exclude the mask from reuse [4, 10, 11].

It is important that each mask is reused by the same HCW who used it in the first place [12]. The name of the mask’s owner (or any other annotation providing identification) must be clearly labeled on the mask. If there is any doubt about who it belongs to or if it is impossible to return the mask to its owner, it should be discarded [11]. This is justified by the risk, despite of being low, of transmitting bacteria, fungi or any other microorganisms between the different HCWs. As the decontamination of masks is not usually practiced, the lack of perspective forces us to be careful.

Some decontamination methods allow the mask to be used more than twice. This obviously involves more than one decontamination cycle. In these cases, in addition to the above criteria, the mask must be correctly annotated with the number of cycles. It must not previously have completed the maximum number of decontamination cycles possible with the specific method of decontamination used. If the maximum decontamination number for a mask is reached, or there is any question about the number of cycles that the mask has undergone, then it should be discarded.

Some articles suggest systematically disposing of all masks that are used by HCWs or in wards that are exposed to SARS-CoV-2 [13]. We are recommending a different course of action for a number of reasons. To begin with, it is likely that the overwhelming majority of N95 or FFP2 masks currently being used in a healthcare facility are used in COVID wards. Throwing them away would therefore not solve the shortage problem. In non-pandemic situations, these types of masks are often used in the presence of diseases such as tuberculosis, that are more difficult to disinfect than SARS CoV-2. In a scenario with limited options, it would therefore be more prudent to decontaminate a mask that is potentially contaminated with SARS CoV-2 than those carrying the mycobacterium causing tuberculosis, for example.

Although generally recommended for medical equipment, is not recommended to clean the masks prior to decontamination, and there are no validated protocols from the manufacturers. The polypropylene filter material loses its hydrophobic layer when wet, meaning that cleaning can affect the filtration properties of the mask [11].

### Step 1: collection

Doffing is a critical step where healthcare professionals could contaminate themselves. It is important that healthcare workers make sure to perform hand hygiene before doffing their mask, and after preparing it for collection. Staff must collect the masks and transport them to the reprocessing site. It is crucial that systems are in place to guarantee their personal safety throughout this activity. In most circumstances, handling these masks with gloves and a mask is sufficient. It is important to note that the composition of the masks and their eligibility for decontamination with the chosen method should be known by the people responsible for infection prevention and environmental hygiene.

The next step is that the used masks which have been deemed eligible for decontamination by their owner are put in a bag or pocket that is compatible with the decontamination system. Each mask has its own pocket (which we will broadly refer to as a “bags”). In the Nebraska study, the personnel put their used masks in plastic bags before they are brought into the UV treatment room [6]. In Switzerland, they are placed in a Tyvek disinfection sheath that has been previously welded on one side or in a Biaxially Oriented Polypropylene (BOPP) plastic bag. In this particular instance, the sleeves were used for sterilization with hydrogen peroxide vapor, while the bags were intended for sterilization by ethylene oxide [14].

Once in their bags, the owners write the date and the serial number on the bag (or other ways to identify them). This must be done with a specific marker, the ink of which is compatible with the decontamination process. In Switzerland, if the bag has two different sides, it is requested that these inscriptions be made on the plastic side and not the paper side [14]. This is because some decontaminating agents may penetrate the paper side, leading to the risk of the ink penetrating and staining the mask. Additionally, the tip of the marker can create tears if used on the paper side, which affects the sterile barrier [15].

The bags are then placed in plastic boxes or similar containers. They are labeled with the names of the wards they belong to as well as the contents (masks to be decontaminated). It is recommended to use different colors for boxes containing the used masks and those containing the decontaminated ones. It is also recommended used and disinfected boxes of masks are stored separately to reduce any risk of cross contamination [14].

Once appropriately bagged, labelled, and put in containers, these containers are transported to the place of storage or decontamination by the staff in charge of the reprocessing. In order to best preserve the integrity of the masks, it is preferable to shorten the storage time as much as possible. The humidity and the presence of microbes form a favorable environment for the proliferation of bacteria and the growth of molds. The presence of mold makes masks ineligible for decontamination, and the must be discarded if any is present [13]. Therefore, efficiency of the transport of the masks is crucial, and it can be advantageous to have a room for drying them.

Once brought to the decontamination area, the personnel in charge must check each mask again according to the same criteria as above (Table 1). Additionally, they should eliminate those with hair on them or with a problem with the inscription (if the inscription missing, illegible, in the wrong place, or made with the wrong marker). In Switzerland, the time required for this task has been estimated at 3 min per mask, including the work sealing the bags [14]. This human and financial cost of the workforce is therefore not negligible.

### Step 2: decontamination

The decontamination of the masks must guarantee that: masks maintain their filtering and mechanical properties after treatment, that the reprocessing procedure destroys the viral load and other potentially pathogenic microorganisms, and that there is no residual toxicity on the mask or in the work area after reprocessing.

The decontamination techniques are detailed in the second part of this article. It is crucial that the system used allows staff to know how many cycles of decontamination a mask has undergone. It should be mandatory that the personnel in charge of decontamination systematically marks the number of cycles performed on a precise location on the mask. This must be done after decontamination and can easily be achieved with a specific marker dedicated to this task. If the space on the mask is limited, one could imagine using other ways of marking the masks compatible with the decontamination process. Before commencing their distribution, the personnel must sort the masks again, and eliminate any which are physically altered or deformed (either on the mask or its support system) or whose number of decontamination cycles are uncertain [12]. An alternative to this method is that the HCWs receiving their masks checks the condition of their mask according to the previous criteria [7].

For healthcare centers that can afford it, it is advisable to regularly test the filtering properties of a sample of individual masks that have undergone the decontamination process. This makes it possible to rapidly detect whether the process is carried out correctly or not.

### Step 3: redistribution

As with their collection, the distribution of the decontaminated masks must be closely monitored. It is also important to store the masks in a dry place [12]. At the end of the decontamination process, the masks of the same caregiver are grouped in a single sterilized plastic bag. Reprocessed masks belonging to the same ward are stored in a plastic case or airtight container (ideally of a different color) than the one containing the worn masks. The box should be labeled with the name of the ward to which it belongs and with its contents. It is advisable if possible to add 30 g of silica gel packets to every 10 masks in the storage boxes in order to keep them as dry as possible [12].

The containers are then transported to the ward and collected by their owners. It goes without saying that the mask must be put on and adjusted by the wearer only after performing proper hand hygiene [2]. An additional fit check could help to identify further potential reprocessing damages.

Communication within the healthcare facility is important as well so that HCWs know where they can go to get their masks and how to inspect them. It is strongly recommended that the masks be nominative and that they therefore return to their former owner, reasons for this are explained in the section “Selection and exclusion criteria” [12].

As such decontamination and reuse of masks is not applied in normal situations, the lack of perspective warrants increased precaution. At HUG, the decontamination pilot project revealed that 27.6% of 341 masks recovered did not complete the cycle [14]. Half of the masks that could not complete the cycle had either an inscription in the wrong place or a stain. Although perfect recycling is impossible and the different methods have their limits, sorting errors must be avoided in order to ensure the efficient function of the circular decontamination system (see Table 1).

### Methods of disinfection and sterilization

The remainder of this article will focus on the analysis of the various methods available for the sterilization and disinfection of masks. Firstly, the difference between decontamination, disinfection and sterilization must be clarified. Decontamination is the neutralization or removal of dangerous substances, radioactivity, or germs from an object, area or person [16]. Disinfection is the antimicrobial reduction of the number of viable micro-organisms to a level previously specified as appropriate for its intended further handling or use [16]. Sterilization is a defined process used to render a surface or product free from viable organisms, including bacterial spores. It also frequently includes the objective of allowing the maintenance of the sterile state [16].

For the sake of clarity and in order to make the comparison easier, the analysis is guided by different criteria and quantified by emojis. We used emojis, because these pictograms can easily be used to transfer concepts and ideas worldwide and they might be conveyed more quickly and effectively than words [17]. Adding emojis to the biomedical literature has been suggested as substitutes for words to augment medical literature. Emojis enable users from different countries to communicate in a standardized way with single compact characters that circumvent language barriers. Using this visual language may be less time-consuming and a way to break down language barriers in the medical literature and make the key ideas in healthcare more accessible to all [18].

It is important to note that when referring to the number studies found in the literature for a specific decontamination method, we are only referring to ones that were included in our systematic review on the subject. This means that only studies that looked at decontaminating N95/FFP2 masks, the microbiological efficacy of a particular method and which had a control were included.

#### Dry heat

This method is quite accessible to healthcare institutions in limited resource environments. Simple dry heat generally results in disinfection, not sterilization, although one study explored a two-step dry heating method that resulted in sterilization [7].

Results were mixed, and generally it is agreed that moist heat has a better microbiological outcome than decontaminating with dry heat. It is possible to use the dry heat cycle of washer-disinfectors for the dry heat sterilization of masks, and one study showed that certain high temperature drying programs are sufficient to deactivate the virus [7]. In another study the authors found that thermal disinfection at 90 °C for 5 min led to important mask deformation [19]. It is important to note that washing programs of those machines may not be used for the masks, as water will degrade them. It may be advantageous to use washer-disinfectors for dry heat mask disinfection, because these machines have a self-decontamination function that drying cabinets or ovens do not have.

#### Ethylene oxide

Ethylene oxide decontamination has shown some promising results with a high log reduction without affecting fit and filtration, and was studied in two experiments in the systematic review (see Table 3) [20, 21]. An ethylene oxide sterilization method (1 h at 55 °C) was tested in a pilot project at HUG [14].

#### Hydrogen peroxide

Gaseous hydrogen peroxide was tested in numerous studies, sometimes in combination with peracetic acid, and often with very successful results (see Table 3). It is used in vapor (HPV) aerosol (aHP) or ionized (HPi) forms. Though there are some differences that could be important when choosing a system for decontaminating large rooms, these are less important in the context of mask decontamination. HPV generally has a higher ppm, higher humidity and longer evaporating time than aerosolized. In HPi, the liquid is ionized, which helps it stick more easily to surfaces once aerosolized. The capacity of masks’ sterilization with gaseous hydrogen peroxide diffusion is strongly demonstrated in the literature. This method is recommended by the Food and Drug Administration in the United States as well as by the Swiss Scientific Task Force regarding COVID-19 [11, 12, 22]. For this type of sterilization, it is important that the masks do not contain cellulose, as it absorbs the hydrogen peroxide, and thus damages the masks’ physical integrity [11]. When this method was tested in a pilot project carried out at HUG, the eligible masks for sterilization were placed by their owners in individual Tyvek disinfection sheaths that had previously been welded on one side.

#### Moist heat (without pressure)

It is important to distinguish between the two distinct methods of moist heat decontamination and steam sterilization. The latter is acquired by steam programs in autoclaves with pressure, while moist heat can be generated by different manners and does not include pressure. Oven generated moist heat, microwave-generated steam and rice cooker moist heat are the treatments considered as “moist heat decontamination” in this paper. Though generally more primitive than steam sterilization, this method is quite accessible to healthcare institutions in limited resource environments.

Decontamination by steam generated in a microwave should be considered because of its high accessibility and its high efficacy [2, 23]. Its presence in the article is therefore justified by its potential use in health centers with limited resources. It is important to note that the metal bars present on certain models of masks for improving fit around the nose may cause sparks if put into a microwave [2]. Numerous studies affirm that no or little changes in mask integrity and fit were observed after exposure [23–25].

##### Ozone sterilization

Ozone is used in a number of disinfection applications in industry, and is known for being very effective, though often causing the degradation of materials [26]. Though there isn’t a large body of literature, some work on this was started during the SARS epidemic in China [27]. Three recent studies in the literature tested the use of ozone. Though microbiological outcomes and fit/filtration results of the facepieces were good, two of the studies showed degradation of the elastic straps that attach to the facepiece of the mask (see Table 3). This method would need to be tested on the specific mask in order to ensure the absence of deformation of the straps.

#### Peracetic acid dry fogging system (PAF)

Only one study in the literature tested this on its own [20], though an additional two used peracetic acid in conjunction with gaseous hydrogen peroxide (see Table 3). Though initial results were very good, this method requires further study. As we have very little data on this method, we did not include it in Table 3, but preferred to list it in this section than in the “Others” section, because the method is quite promising.

#### Steam sterilization (with pressure)

Steam sterilization refers to sterilization with saturated water vapor at different lengths of time and temperatures with a pre-vacuum. Three studies looked at it in the literature used for the systematic review, and results were quite mixed (see Table 3). Though this method is approved by some European national bodies, others argue that this method may alter the filtering properties of masks and affect their integrity vary according to their quality [7, 28]. This method works better on some types of masks than others [4]. The infrastructure needed for this type of disinfection is already in place in some institutions, as vaporization is sometimes used for the disinfection of mattresses [7].

#### Ultraviolet germicidal irradiation (UVGI)

The method of disinfection by exposure to UV-C rays was the first method of decontamination studied as a response to the respirator shortage in the COVID-19 pandemic [6]. We found over 18 studies that tested UVGI, two in conjunction with heat (see Table 3). Results were mixed, though one study did reach sterilization [29].

## Others

Other sterilization and disinfection methods are discussed in the literature. We did not analyze them in detail in this article for the following reasons:

*Alcohol disinfection *NOT RECOMMENDED—reduces the filtering capacity of masks [11].

*Benzalkonium Chloride disinfection* NOT RECOMMENDED—reduces the filtering capacity of masks [30].

*Chlorine disinfection* NOT RECOMMENDED—reduces the filtering capacity of the masks though possibly not badly enough to go below acceptable levels [8, 11]. More importantly, chlorine residues on the masks endanger the health of the HCWs [3, 4, 31]. One study concluded that further research with lower concentrations of chlorine chemical methods for neutralizing residuals would be worth looking into [3].

*Dry microwave oven irradiation* NOT RECOMMENDED—can partially melt the masks and thus not recommended without modifications [31]. Dry microwave oven irradiation needs improvement before decontamination and subsequent reuse.

*Formaldehyde gas *NOT RECOMMENDED—Though it has been tested in an unpublished study referenced in a white paper [32], formaldehyde is known to fix proteins, which would exclude it form use in most institutions due to concerns about prion diseases.

*Gamma irradiation* NOT RECOMMENDED—may affect the electrostatic properties of the mask fibers and reduce their filtering capacity [33, 34].

*UV-A disinfection* NOT RECOMMENDED—not microbiologically effective [35].

*Washing masks or using detergent* NOT RECOMMENDED—There are no validated protocols from the manufacturers. Washing with soapy water may affect the electrostatic properties of the mask fibers or even deform them [36].

## Limitations

It should be noted that the proposed solution of reusing masks designed for single use would not be considered outside of the context of a health crisis, and that the selection of the criteria used was to a certain extent arbitrary. The two main concerns are the legal implications of reprocessing single use material and the safety of doing so. The scientific literature is poor relating to this subject, and different models of masks may react differently to decontamination. Much of the work studying disposable mask reuse has been conducted as quickly as possible in real-life contexts to address an urgent need as safely as possible. Although a few preliminary summaries of the findings have been proposed, this field of research is in full expansion because of the crisis, and today’s knowledge is rapidly evolving. The data in this article is therefore not exhaustive. It only reflects the information we have at present, and tries to make recommendations to the best of the current knowledge.

One major issue that we did not explore is institutions’ reprocessing capacity. At HUG, at the height of the pandemic, approximately 3000 FFP2 masks were used every day—a number that is not easy to reprocess with the internal capacity available, regardless of the method.

The goal of this paper is to facilitate the understanding of the challenges and benefits of the different methods, not to prioritize one method over another. In this way, we aim to help healthcare facilities choose the methods best suited to their individual contexts and available resources.

It is important to highlight that if microbiological results in the literature vary significantly for a particular method, then it doesn’t necessarily mean that the method is bad. The experiments we analyzed for our systematic review were of varying quality, using different protocols, strengths of intervention (UV dose/ppm/ temperature, etc.) as well as different test organisms.

Our analysis focuses primarily on the practical aspects of implementing the methods of decontamination, rather than the intrinsic functions of said methods. It is therefore important to highlight that the implementation of a mask decontamination system is the responsibility of the facility in which it is implemented. We recommend that institutions contact the manufacturers of the masks they use before setting up a mask decontamination system [2].

## Conclusions

Regarding the Covid-19 pandemic, it is reasonable to say that no single intervention will be able to avoid mask shortage at a moment when demand is exponential and supply chains are disrupted. This challenge must associate industrials as the European EN 149 standard already allows the multiple use if validated by the manufacturer. Some countries, such as the USA [37] have official national recommendations for reprocessing solutions and factsheets to help healthcare professionals balance the benefits and the risks of such a strategy, and others are undoubtedly in the works. Leaving all the decisions and choices at the institutional level is risky, as not all the settings have the same ability for managing such a complex process.

All of the analyzed methods have their advantages and disadvantages, depending on the resources, priorities and individual environments of the healthcare facilities looking to implement them. Ultraviolet irradiation, hydrogen peroxide vapor, and steam sterilization have been recommended by the CDC as promising methods of mask decontamination, while hydrogen peroxide and ethylene oxide are the preferred methods for disposable mask decontamination of the Swiss Society of Hospital Sterilization [2, 4]. Though this may be true for some facilities’ environments, it is certainly not true or even possible for all of them. The more limited the resources are in a healthcare environment, the higher the likelihood that a facility will implement a simple or low-cost solution. Our review provides a basic overview of the different methods and does not mean to be a totally exhaustive resource. We encourage healthcare facilities to contact local experts as well as the manufacturers of their masks and decontamination equipment that they may have at their disposal for additional guidance and information.

## Abbreviations

aHP: Aerosolized hydrogen peroxide
COVID-19: Coronavirus disease 2019
FFP2: Filtering facepiece 2
HCW: Healthcare worker
HPi: Ionized hydrogen peroxide
HPV: Hydrogen peroxide vapor
HUG: University Hospitals of Geneva
IPC: Infection prevention and control
N95: A National Institute for Occupational Safety and Health-certified respirators with a 95% filtration capacity
PAF: Peracetic acid dry fogging system
SARS CoV-2: Severe acute respiratory syndrome coronavirus 2
SSSH: Swiss Society for Hospital Sterilization
UV: Ultraviolet
UVA: Ultraviolet-A
UVGI: Ultraviolet germicidal irradiation
